# Supplementary material for: Antiskyrmions stabilized at interfaces by anisotropic Dzyaloshinskii-Moriya interactions
Source: Nat Commun. 2017 Aug 21;8:308. doi: 10.1038/s41467-017-00313-0 (PMC5566362; doi:10.1038/s41467-017-00313-0)
Supplement: Supplementary file 1 — Supplementary Information [file 41467_2017_313_MOESM1_ESM.pdf]

File name: Supplementary Information
Description: Supplementary Figures, Supplementary Notes, Supplementary Table and Supplementary References.

File name: Supplementary Movie 1
Description: Skyrmion and Antiskyrmion.

File name: Supplementary Movie 2
Description: Multipole expansion of an Antiskyrmion.

File name: Peer Review File
Description:

Supplementary Note 1 | Mathematical criteria for optimality of antiskyrmions and coexistence with skyrmions

In this Supplementary Note we rigorously identify simple criteria on the spiralization tensor that predict the energetic optimality of antiskyrmions versus skyrmions (Theorem 1) and the coexistence of skyrmions and antiskyrmions (Theorem 3) under the influence of a sufficiently large Zeeman field, respectively.

Theorem 1 *Suppose the spiralization tensor has negative determinant. Then for sufficiently large Zeeman field, the least energy over all non-trivial homotopy classes is necessarily attained by an antiskyrmion.*

The key is to use independent orthogonal transformations in spin space *and* real space in order to bring DMI to a canonical form, which allows to utilize arguments from ref. 1 showing that the skyrmion is favoured for the cubic Hamiltonian $D\,\mathbf{m}\cdot\nabla\times\mathbf{m}$. The necessary extension to the case of anisotropic DMI will be provided in Theorem 2 below.

Relative skyrmion number. The notion of skyrmion/antiskyrmion refers to a local energy minimizer within the topological class characterized by the relative skyrmion number $N=\pm 1$, respectively. For sufficiently regular fields $\mathbf{m}=(m_x,m_y,m_z)$ decaying to the background state $m_z(\infty)=\pm 1$, the index N is defined relative to this background state, i.e.

$$N(\mathbf{m}) = -m_z(\infty)Q(\mathbf{m}), \quad (1)$$

where Q is the conventional topological charge

$$Q(\mathbf{m}) = \frac{1}{4\pi}\int_{\mathbb{R}^2} \mathbf{m}\cdot(\partial_x\mathbf{m}\times\partial_y\mathbf{m})\,d\mathbf{r}. \quad (2)$$

In a typical situation where the horizontal magnetization field vanishes at a single point (skyrmion center) the relative skyrmion number N agrees with the index of the horizontal magnetization field at the skyrmion center. It is customary to fix the background state $m_z(\infty)=1$, which leads to the characterization $Q=-1$ for skyrmions and $Q=+1$ for antiskyrmions.

General form of DMI. We consider energy densities

$$e_{\mathrm{DM}}(\mathcal{D};\mathbf{m}) = \sum_\nu \mathbf{D}_\nu\cdot(\partial_\nu\mathbf{m}\times\mathbf{m}), \quad (3)$$

where $\mathbf{D}_\nu$ with $\nu=x,y,z$ are the (micromagnetic) DM vectors, the columns of the spiralization tensor $\mathcal{D}=(\mathcal{D}_{\mu\nu})\in\mathbb{R}^{3\times 3}$, i.e. $\mathcal{D}_{\mu\nu}$ is the μ-th component of the ν-th DM vector. For 3D systems, the chirality tensor

$$\mathcal{L}(\mathbf{m}) = \nabla\mathbf{m}\times\mathbf{m}, \quad (4)$$

whose components are the Lifshitz invariants of $\mathbf{m}$, can then be used to write DMI in form of a matrix inner product

$$e_{\mathrm{DM}}(\mathcal{D};\mathbf{m}) = \mathcal{D}:\mathcal{L}(\mathbf{m}) = \sum_{\mu,\nu}\mathcal{D}_{\mu\nu}\mathcal{L}_{\mu\nu}(\mathbf{m}). \quad (5)$$

For 2D systems with $\mathbf{D}_\nu\perp\hat{\mathbf{e}}_z$ for $\nu=x,y$, the spiralization and chirality tensors may be reduced to $\mathbb{R}^{2\times 2}$ matrices, respectively. We shall adopt the same notation for the reduced tensors $\mathcal{D}\in\mathbb{R}^{2\times 2}$ and $\mathcal{L}(\mathbf{m})\in\mathbb{R}^{2\times 2}$ given by

$$\mathcal{L}_{\mu\nu}(\mathbf{m}) = \sum_\kappa \epsilon_{\mu\kappa}\left(m_z\,\partial_\nu m_\kappa - m_\kappa\,\partial_\nu m_z\right). \quad (6)$$

Here and in what follows greek indices denote in-plane Cartesian coordinates x,y, and $\epsilon_{\mu\nu}$ and $\delta_{\mu\nu}$ denote Levi-Civita and Kronecker symbols, respectively. For spiralization tensors $\mathcal{D}\in\mathbb{R}^{2\times 2}$, formula (5) features a general micromagnetic form of interface-induced DMI. It also includes the 2D reduction of cubic DMI $\mathcal{D}_{\mu\nu}=D\,\delta_{\mu\nu}$ differing from the prototypical thin-film DMI $\mathcal{D}_{\mu\nu}=D\,\epsilon_{\mu\nu}$ only by a rigid rotation in spin space.

Canonical form of DMI. Given orthogonal transformations $\mathcal{R},\mathcal{S}\in O(2)$ in real space and horizontal spin space, respectively, we represent $\mathbf{m}(\mathbf{r})=\mathcal{S}\tilde{\mathbf{m}}(\tilde{\mathbf{r}})$ with $\tilde{\mathbf{r}}=\mathcal{R}\mathbf{r}$. Then, the topological charge satisfies

$$Q(\mathbf{m}) = \det(\mathcal{R}\mathcal{S})\,Q(\tilde{\mathbf{m}}). \quad (7)$$

The chirality tensor satisfies

$$\mathcal{L}(\mathbf{m}) = (\det\mathcal{S})\,\mathcal{S}\mathcal{L}(\tilde{\mathbf{m}})\mathcal{R}, \quad (8)$$

to be evaluated at $\mathbf{r}$ and $\tilde{\mathbf{r}}$, respectively. Defining the auxiliary spiralization tensor $\tilde{\mathcal{D}}$ by

$$\mathcal{D} = (\det\mathcal{S})\,\mathcal{S}\tilde{\mathcal{D}}\mathcal{R}, \quad (9)$$

it follows from (5) that

$$\int_{\mathbb{R}^2} e_{\mathrm{DM}}(\mathcal{D};\mathbf{m})\,d\mathbf{r} = \int_{\mathbb{R}^2} e_{\mathrm{DM}}(\tilde{\mathcal{D}};\tilde{\mathbf{m}})\,d\tilde{\mathbf{r}}. \quad (10)$$

Since Heisenberg exchange, uniaxial anisotropy and Zeeman interaction along the vertical direction in spin space are invariant with respect to the transformation between $\mathbf{m}$ and $\tilde{\mathbf{m}}$, the corresponding transformation between $\mathcal{D}$ and $\tilde{\mathcal{D}}$ serves as a reduction to a canonical problem. In fact, by virtue of a singular value decomposition we can always achieve $\tilde{\mathcal{D}}$ to be diagonal and positive semidefinite, leading to the canonical form of DMI

$$e_{\mathrm{DM}}(\tilde{\mathcal{D}};\tilde{\mathbf{m}}) = D_1\mathcal{L}_1(\tilde{\mathbf{m}}) + D_2\mathcal{L}_2(\tilde{\mathbf{m}}), \quad (11)$$

where $0\le D_1\le D_2$ are the singular values of $\mathcal{D}$ and $\mathcal{L}_1=\mathcal{L}_{xx}$ and $\mathcal{L}_2=\mathcal{L}_{yy}$ the diagonal elements of $\mathcal{L}$.

Note that $|\mathcal{D}|^2 = D_1^2 + D_2^2$, where $|\mathcal{D}| = \sqrt{\mathcal{D} : \mathcal{D}}$ denotes the Frobenius norm of $\mathcal{D}$.

In order to prove Theorem 1, we shall argue that in the nondegenerate case $D_1 > 0$ the transformed problem with DMI (11) selects skyrmions ($Q = -1$). It then follows from (7) that in the original problem with spiraliation tensor $\mathcal{D}$ antiskyrmions are selected provided $\det(\mathcal{R}\mathcal{S}) = -1$, i.e. if $\det \mathcal{D} < 0$. Theorem 1 is therefore a consequence of the following:

Theorem 2 *Suppose the spiralization tensor is diagonal and positive definite. Then for sufficiently large Zeeman field, the least energy over all non-trivial homotopy classes is necessarily attained by a skyrmion.*

Let us sketch the energy bounds leading to the proof of Theorem 2. Dropping the tilde notation we let

$$e_{\mathrm{DM}}(\mathbf{m}) = D_1 \mathcal{L}_1(\mathbf{m}) + D_2 \mathcal{L}_2(\mathbf{m}) \tag{12}$$

and consider the micromagnetic energy functional

$$E(\mathbf{m}) = \int_{\mathbb{R}^2} \frac{J}{2} |\nabla \mathbf{m}|^2 + e_{\mathrm{DM}}(\mathbf{m}) + B(1 - m_z) + K_\perp (m_z^2 - 1)\, \mathrm{d}\mathbf{r}. \tag{13}$$

for $B \geq 0$ and $K_\perp \leq 0$.

Upper energy bounds.

$$E_Q < 4\pi J \quad \text{for} \quad Q = \pm 1. \tag{14}$$

For this qualitative estimate, which is only sharp in the large field limit, it is sufficient to take into account equivariant (axisymmetric) configurations. Constructing trial profiles for skyrmion cores in the topological class $Q = -1$ with minimal Heisenberg exchange of $4\pi J$ amounts to a modification of the stereographic map, well-establised in the framework of Belavin-Polyakov solitons[2]. With $\mathbf{r}^\perp = (-r_y, r_x)$, we specifically choose

$$\mathbf{m}_{\mathrm{core}}(\mathbf{r}) = \left(\frac{2\mathbf{r}^\perp}{r^2 + 1}, \frac{r^2 - 1}{r^2 + 1} \right), \tag{15}$$

featuring a counter-clockwise curling of the horizontal components as preferred by (11). We have $\int_{\mathbb{R}^2} \mathcal{L}_1(\mathbf{m}_{\mathrm{core}})\, \mathrm{d}\mathbf{r} = \int_{\mathbb{R}^2} \mathcal{L}_2(\mathbf{m}_{\mathrm{core}})\, \mathrm{d}\mathbf{r} = -4\pi$, thus

$$\int_{\mathbb{R}^2} e_{\mathrm{DM}}(\mathbf{m}_{\mathrm{core}})\, \mathrm{d}\mathbf{r} = -(D_1 + D_2) 4\pi. \tag{16}$$

A trial profile of opposite charge is obtained by reflection in horizontal spin space. For $\overline{\mathbf{m}} = (m_x, -m_y, m_z)$, we have $Q(\overline{\mathbf{m}}) = -Q(\mathbf{m})$ while $\mathcal{L}_1(\overline{\mathbf{m}}) = -\mathcal{L}_1(\mathbf{m})$ and $\mathcal{L}_2(\overline{\mathbf{m}}) = \mathcal{L}_2(\mathbf{m})$. We obtain

$$\int_{\mathbb{R}^2} e_{\mathrm{DM}}(\overline{\mathbf{m}}_{\mathrm{core}})\, \mathrm{d}\mathbf{r} = (D_1 - D_2) 4\pi. \tag{17}$$

while the other energy contributions remain unchanged. Zeeman and anisotropy energies diverge logarithmically for (15). But using a cut-off and scaling argument to balance all energy contributions (see ref. 1), one deduces (14) provided $D_1 < D_2$. The bound holds independently of B but narrows down for increasing B. In case of equality $D_1 = D_2$ treated in ref. 1, (14) holds only for $Q = -1$.

Ansatz-free lower bounds. For $BJ/|\mathcal{D}|^2 \geq 2$ the energy is non-negative and

$$E_Q > E_1 > E_{-1} \quad \text{for all} \quad |Q| > 1. \tag{18}$$

A straight forward lower energy bound is obtained by bounding the absolute value of DMI in terms of exchange and Zeeman interaction. The argument is independent of the specific form of DMI $e_{\mathrm{DM}}(\mathbf{m}; \mathcal{D}) = \mathcal{D} : \mathcal{L}(\mathbf{m})$ and only depends on its size $|\mathcal{D}|$. Using the translation invariance of DMI in (vertical) spin space, i.e.

$$\mathcal{D} : \mathcal{L}(\mathbf{m}) = \mathcal{D} : \mathcal{L}(\mathbf{m} - \hat{\mathbf{e}}_z) + \text{boundary terms}, \tag{19}$$

and $|\mathcal{D} : \mathcal{L}(\mathbf{m} - \hat{\mathbf{e}}_z)| \leq |\mathcal{D}||\nabla \mathbf{m}||\mathbf{m} - \hat{\mathbf{e}}_z|$, we obtain from the elementary inequality $|ab| \leq \frac{1}{2}(a^2 + b^2)$

$$\left| \int_{\mathbb{R}^2} e_{\mathrm{DM}}(\mathbf{m}; \mathcal{D})\, \mathrm{d}\mathbf{r} \right| \leq \int_{\mathbb{R}^2} \tfrac{|\mathcal{D}|^2}{2B} |\nabla \mathbf{m}|^2 + \tfrac{B}{2} |\mathbf{m} - \hat{\mathbf{e}}_z|^2\, \mathrm{d}\mathbf{r}. \tag{20}$$

Taking into account that $|\mathbf{m} - \hat{\mathbf{e}}_z|^2 = 2(1 - m_z)$, it follows by virtue of the classical topological lower bound

$$\frac{1}{2} \int_{\mathbb{R}^2} |\nabla \mathbf{m}|^2\, \mathrm{d}\mathbf{r} \geq 4\pi |Q(\mathbf{m})|, \tag{21}$$

cf. Belavin and Polyakov[2], that

$$E(\mathbf{m}) \geq 4\pi \left(J - \tfrac{|\mathcal{D}|^2}{B} \right) |Q(\mathbf{m})|. \tag{22}$$

Accordingly, we infer $E_Q > E_{\pm 1}$ for $|Q| > 1$ and $BJ/|\mathcal{D}|^2 \geq 2$. It remains to show that $E_{-1} < E_1$. To this end, we shall modify the Bogomolny type lower bound $E(\mathbf{m}) \geq 4\pi J Q(\mathbf{m})$ valid for all $\mathbf{m}$ with $Q(\mathbf{m}) \geq 0$ provided $D_1 = D_2 \neq 0$ and $BJ/D_2^2 \geq 1$ (see ref. 1). The bound extends to the case $0 \leq D_1 < D_2$ as

$$E(\mathbf{m}) \geq 4\pi J Q(\mathbf{m}) - (D_2 - D_1) \int_{\mathbb{R}^2} \mathcal{L}_1(\mathbf{m})\, \mathrm{d}\mathbf{r}. \tag{23}$$

We shall argue by contradiction: Suppose $E_1 < E_{-1}$ and $\mathbf{m}^{(k)}$ is a minimizing sequence with $Q(\mathbf{m}^{(k)}) = 1$ and $E(\mathbf{m}^{(k)}) \to E_1$ as $k \to \infty$. Then it follows from (14) and (23) with $Q = 1$ that $\int_{\mathbb{R}^2} \mathcal{L}_1(\mathbf{m}^{(k)})\, \mathrm{d}\mathbf{r} \geq \lambda$ for some $\lambda > 0$ as $k \to \infty$. But for the reflected fields $\overline{\mathbf{m}}^{(k)}$ (cf. discussion preceding (16)) we have $E_{-1} \leq E(\overline{\mathbf{m}}^{(k)}) \leq E(\mathbf{m}^{(k)}) - 2D_1\lambda < E_1$ as $k \to \infty$, a contradiction.

Attainment of $E_{\pm 1}$. Energy upper and lower bounds can be used to rule out possible scenarios of non-attainment of $E_{\pm 1}$ by virtue of the concentration-compactness method (see e.g. ref. 3 in the context of the classical Skyrme model). For $0 < D_1 = D_2$ we have $E_Q < 4\pi J$ only for $Q \in \{0, -1\}$, which yields attainment only for $Q = -1$, see ref. 1. Let us now assume $0 \leq D_1 < D_2$. The bounds (14) and (18) then imply

$$E_{\pm 1} < E_{-Q} + E_{(Q\pm 1)} \quad \text{for all} \quad |Q| > 1, \qquad (24)$$
$$E_{\pm 1} < 4\pi J|Q| + E_{(Q\pm 1)} \quad \text{for all} \quad |Q| > 0. \qquad (25)$$

Inequality (24) rules out the splitting into two well-separated field configurations of non-vanishing topological charge (dichotomy). Inequality (25) rules out the gain or loss of topological charge by concentration effects. In fact, the formation of one or more point singularities of total charge $Q \neq 0$ amounts to an energy of at least $4\pi J|Q|$ (see the discussion following (14)). Ruling out the vanishing case by means of (14) and a Sobolev inequality as in ref. 1, we have exhausted all possible scenarios of non-attainment and obtain:

Theorem 3 *Suppose the spiralization tensor admits different singular values. Then skymions and antiskyrmions coexist for large enough Zeeman field.*

Supplementary Note 2 | Multipole expansion of an Antiskyrmion

In the main text we stated that an antiskyrmion can be understood as an addition of a quadrupolar field to the monopole of the skyrmion. This statement will be substantiated in this Supplementary Note. We will show in the following the multipole expansion of the magnetization field of the antiskyrmion on a sphere (see Supplementary Fig. **1**(a)) in terms of the vector spherical harmonics which are defined as[4]

$$\mathbf{Y}_{lm} = Y_{lm}\,\hat{\mathbf{e}}_{\mathbf{r}}$$
$$\mathbf{\Psi}_{lm} = r\nabla Y_{lm}$$
$$\mathbf{\Phi}_{lm} = \hat{\mathbf{e}}_{\mathbf{r}} \times \nabla Y_{lm}\,. \qquad (26)$$

It is obvious, that a hedgehog-type chiral skyrmion as shown in Fig. 1(a) of the main text has the monopole ($\mathbf{Y}_{00}$) as the only non-vanishing component with an expansion coefficient $c_{00}^{\mathbf{Y}} = M\sqrt{4\pi}$, with $M = |\mathbf{m}(\mathbf{R})|$, being the absolute value of the magnetization. For the antiskyrmion, however, this monopole contribution is reduced ($c_{00}^{\mathbf{Y}} = \frac{1}{3}\, M\sqrt{4\pi}$) and additionally quadrupolar contributions arise. The resulting expansion coefficients are $c_{20}^{\mathbf{Y}} = \frac{\sqrt{20}}{15}\, M\sqrt{4\pi}$, $c_{2-2}^{\mathbf{Y}} = c_{22}^{\mathbf{Y}} = -\frac{\sqrt{30}}{15}\, M\sqrt{4\pi}$, $c_{20}^{\mathbf{\Psi}} = \frac{\sqrt{5}}{15}\, M\sqrt{4\pi}$, and $c_{2-2}^{\mathbf{\Psi}} = c_{22}^{\mathbf{\Psi}} = -\frac{\sqrt{30}}{30}\, M\sqrt{4\pi}$. The non-vanishing multipole components are visualized in Supplementary Fig. **1**, where the length of the arrows scales with the expansion coefficient.

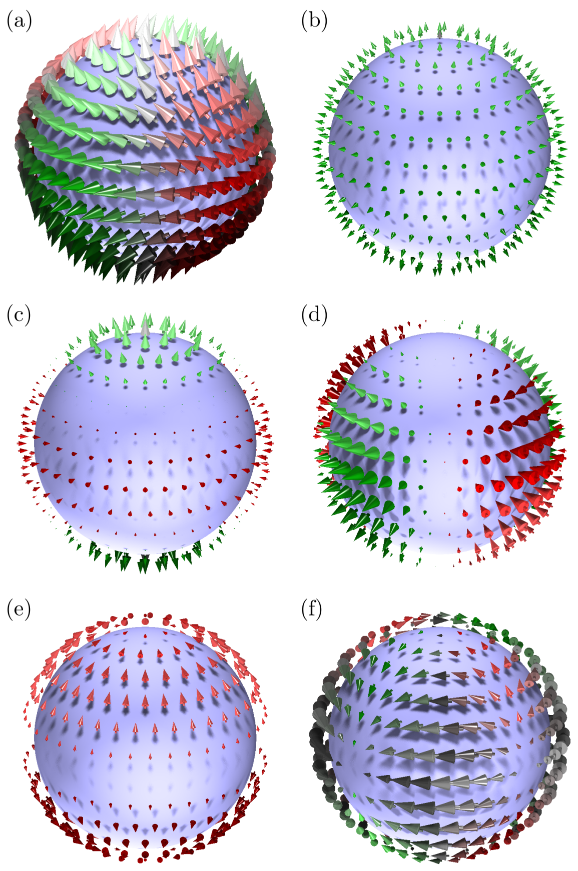

Supplementary Figure 1 | Visualization of the multipole expansion of an antiskyrmion. Non-zero contributions of the multipole expansion of the antiskyrmion shown in (a): (b) $\mathbf{Y}_{00}$, (c) $\mathbf{Y}_{20}$, (d) $\mathbf{Y}_{2-2} + \mathbf{Y}_{22}$, (e) $\mathbf{\Psi}_{20}$, and (f) $\mathbf{\Psi}_{2-2} + \mathbf{\Psi}_{22}$. The length of the arrows indicates the size of the expansion coefficients (see text).

Supplementary Note 3 | Magnetostatic energy of axisymmetric skyrmions vs. antiskyrmions.

In non-dimensionalized form, the averaged magnetostatic energy $E_{\rm mag}(\mathbf{m})$ induced by a magnetization distribution $\mathbf{m}$ on an infinite film of thickness t is given by

$$\frac{1}{8\pi t}\int_{\mathbb{R}^3}\int_{\mathbb{R}^3} \frac{(\nabla\cdot\mathbf{m})(\mathbf{r})(\nabla\cdot\mathbf{m})(\mathbf{r}')}{|\mathbf{r}-\mathbf{r}'|}\mathrm{d}\mathbf{r}\mathrm{d}\mathbf{r}', \qquad (27)$$

where $\nabla\cdot\mathbf{m}$ is the distributional divergence of $\mathbf{m}$ extended by zero outside the film. If $\mathbf{m}$ is assumed to be z-independent inside the film, the contributions to $E_{\rm mag}(\mathbf{m})$ from m_z and $m_\parallel$ separate. To leading orders in t, this gives rise to a decomposition $E_{\rm mag}(\mathbf{m}) \approx E_{\rm mag}^{\perp}(m_z) + E_{\rm mag}^{\parallel}(\sigma)$ into a shape anisotropy part

$$E_{\rm mag}^{\perp}(m_z) = \frac{1}{2}\int_{\mathbb{R}^2} m_z^2\mathrm{d}\mathbf{r} \qquad (28)$$

and a film charge part

$$E^{\parallel}_{\text{mag}}(\sigma) = \frac{t}{8\pi}\int_{\mathbb{R}^2}\int_{\mathbb{R}^2}\frac{\sigma(\mathbf{r})\sigma(\mathbf{r}')}{|\mathbf{r}-\mathbf{r}'|}\mathrm{d}\mathbf{r}\mathrm{d}\mathbf{r}' \tag{29}$$

in terms of the in-plane divergence $\sigma(\mathbf{r}) = (\nabla\cdot m_{\parallel})(\mathbf{r})$. This thin film reduction[5] can conveniently be derived from a Fourier space representation, in which the film charge part admits the form

$$E^{\parallel}_{\text{mag}}(\sigma) = \frac{t}{4}\int_{\mathbb{R}^2}\frac{|\hat{\sigma}(\mathbf{k})|^2}{|\mathbf{k}|}\mathrm{d}\mathbf{k}. \tag{30}$$

We shall estimate the contribution of skyrmions and antiskrymions to $E_{\text{mag}}(\mathbf{m})$ considering the standard axisymmetric ansatz with magnetization densities of the form[6,7]

$$\mathbf{m}(\mathbf{r}) = \begin{pmatrix}\cos\Phi(\varphi)\sin\theta(\rho)\\ \sin\Phi(\varphi)\sin\theta(\rho)\\ \cos\theta(\rho)\end{pmatrix} \tag{31}$$

with cylindrical coordinates (ρ,φ) in real and polar coordinates (θ,Φ) in spin space, depending on ρ and φ, respectively. The phase function has the form

$$\Phi(\varphi) = v\varphi + \gamma \tag{32}$$

with winding number $v = \pm 1$ to distinguish skyrmions from antiskyrmions, and a phase shift γ to tune chirality according to the chirality vector $\mathbf{c}_\chi$. With the left-handed Néel-type skyrmion $\mathbf{m}_{\text{Néel}}$ (with $v = 1$ and $\gamma = 0$) as reference configuration, an arbitrary $\mathbf{m}$ of the form (31) is obtained by an orthogonal transformation in horizontal spin space, i.e.

$$\mathbf{m} = \mathcal{S}\mathbf{m}_{\text{Néel}} \quad \text{for some} \quad \mathcal{S}\in O(2), \tag{33}$$

keeping shape anisotropy invariant. With in-plane divergences of $\mathbf{m}$ and $\mathbf{m}_{\text{Néel}}$ denoted by σ and $\sigma_{\text{Néel}}$, respectively, it follows from a symmetry argument in (30) that

$$\frac{E^{\parallel}_{\text{mag}}(\sigma)}{E^{\parallel}_{\text{mag}}(\sigma_{\text{Néel}})} = \frac{1}{2\pi}\int_{\{|\mathbf{k}|=1\}}|\mathbf{k}\cdot(\mathcal{S}\mathbf{k})|^2\mathrm{d}\mathbf{k}. \tag{34}$$

Expressed in terms of v and γ (34) reads

$$\frac{E^{\parallel}_{\text{mag}}(\sigma)}{E^{\parallel}_{\text{mag}}(\sigma_{\text{Néel}})} = \begin{cases}\cos^2\gamma \text{ for skyrmions } v=1,\\ \frac{1}{2} \text{ for antiskyrmions } v=-1.\end{cases} \tag{35}$$

Accordingly, the effect of film charge differs significantly for different kinds of chiral skyrmions. It is minimal (zero) for Bloch-type ($\gamma = \pm\frac{\pi}{2}$), and maximal for Néel-type skyrmions ($\gamma = 0,\pi$). Axisymmetric antiskyrmions are precisely in the middle with a value independent of γ. The argument may be extended to the case of anisotropic DMI and almost axisymmetric (anti-)skyrmions near the conformal high energy limit[8] $JB/|\mathcal{D}|^2 \gg 1$.

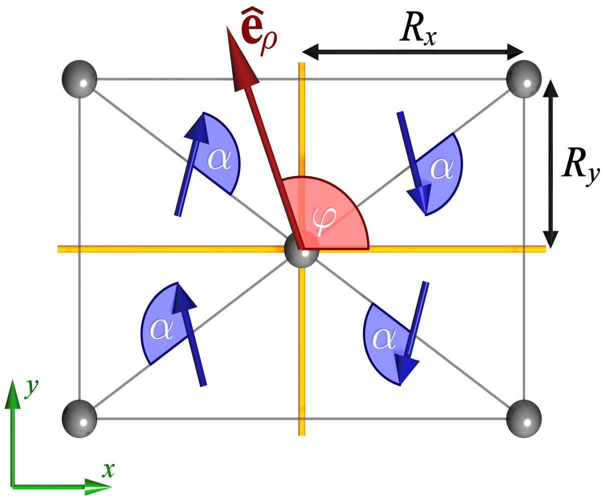

Supplementary Figure 2 | Visualization of the investigated geometry. Visualization of the angles and distances introduced in the text for the description of a system with C_{2v} symmetry.

Supplementary Note 4 | Multichirality in systems with the C_{2v} symmetry from a micromagnetic analysis

In the main paper we have shown that for surfaces or interfaces with C_{2v} symmetry, the symmetry is so low that the in-plane component of the DMI vector, $\hat{\mathbf{e}}_{\parallel\text{DM}}$, is not symmetry-determined, but can take on any in-plane direction depending on details of the electronic structure of the system. We had selected two particular directions of $\hat{\mathbf{e}}_{\parallel\text{DM}}$, one resulting in a skyrmion and one in an antiskyrmion. In this supplementary note, we generalize the equations to arbitrary in-plane directions and to any system with a C_{2v} symmetry.

In a system with C_{2v} symmetry, the general form of the spiralization tensor reads[9]

$$\mathcal{D} = \begin{pmatrix} 0 & \mathcal{D}_{12}\\ \mathcal{D}_{21} & 0\end{pmatrix}, \tag{36}$$

where $\mathcal{D}_{12}$ and $\mathcal{D}_{21}$ contain all information about the geometry of the lattice and result from the summation over all neighbours. Note, that $\det\mathcal{D} = -\mathcal{D}_{12}\mathcal{D}_{21}$ and $\mathcal{D}_{\mu\nu}$, $\mu\neq\nu$, can be related to the singular values of the Supplementary Note 1 by $D_{1(2)} = \min(\max)(|\mathcal{D}_{12}|,|\mathcal{D}_{21}|)$. It is insightful to map the spiralization tensor (36) onto a nearest-neighbour model (see Supplementary Fig. **2**) with an effective n.n. DM vector, $\mathbf{D}_{\text{nn}}$. The two linearly independent components of $\mathbf{D}_{\text{nn}} = (D_x, D_y)$, translate into two independent parameters of the spiralization tensor, which becomes

$$\mathcal{D} = \frac{1}{A_\Omega}\begin{pmatrix} 0 & 4D_xR_y\\ 4D_yR_x & 0\end{pmatrix}. \tag{37}$$

This spiralization tensor is obviously equivalent to (36) with $\mathcal{D}_{12} = 4D_xR_y/A_\Omega$ and $\mathcal{D}_{21} = 4D_yR_x/A_\Omega$ and is therefore capable to describe the identical mathematical problem. We express the direction of the microscopic DM vector,

$$\mathbf{D}_{\rm nn} = \begin{pmatrix} D_x \\ D_y \end{pmatrix} = \frac{Ð}{R}\begin{pmatrix} \cos\alpha\ R_x + \sin\alpha\ R_y \\ -\sin\alpha\ R_x + \cos\alpha\ R_y \end{pmatrix}, \quad (38)$$

relative to the vector of the chemical bond connecting the atom at site $\mathbf{R}_0$ and the n.n. sites $\mathbf{R}_{\rm nn}$, $\mathbf{R}_{\rm nn} = (R_x, R_y)$, in terms of angle α, where $Ð = \sqrt{D_x^2 + D_y^2}$ and $R = \sqrt{R_x^2 + R_y^2}$ and $1/A_\Omega$ is the area of the surface unit cell. Hence, the angles $\alpha = \pi/2$ and π reproduce the spiralization tensors discussed in the main text. The introduced angles and the lattice parameters are visualized in Supplementary Fig. **2**.

According to the main text, the Néel-type chirality, $\mathcal{C}_{\rm N}$, for a magnetic winding along direction $\hat{\mathbf{e}}_\rho = (\cos\varphi, \sin\varphi)^{\rm T}$ is then given by

$$\begin{aligned}\mathcal{C}_{\rm N} &= A_\Omega(\mathcal{D}\hat{\mathbf{e}}_\rho)_\varphi \\ &= \frac{4\,Ð}{R}R_xR_y\cos\alpha\ \cos(2\varphi) \\ &\quad -\frac{4\,Ð}{R}R_xR_y\sin\alpha\left(\cos^2\varphi\ \frac{R_x}{R_y} + \sin^2\varphi\ \frac{R_y}{R_x}\right) \quad (39)\end{aligned}$$

For a fixed angle α, only the first term $\propto \cos 2\varphi$ can change the sign of the chirality $\mathcal{C}_{\rm N}(\varphi,\alpha)$ as function of φ, while the second term supports monochirality. Hence, α weights the relative competition between multi- and monochirality, or antiskyrmion and skyrmion formation, respectively.

We now restrict ourselves to the geometry of a bcc(110) surface, for which $R_x = a/\sqrt{2}$ and $R_y = a/2$, with a being the lattice parameter. Analysing $\mathcal{C}_{\rm N}(\varphi,\alpha)$ as a function of the direction of the in-plane DM vector in terms of α and the direction of $\hat{\mathbf{e}}_\rho$ parametrized by φ, we see in Supplementary Fig. **3** that the DM-field changes sign as function of φ for a broad range of α, i.e. $-\arctan(\sqrt{2}) < \alpha < \arctan(1/\sqrt{2})$, thus favouring magnetization fields of multichiral character. Outside this parameter range of α we have monochiral DM-fields either of positive ($\alpha > \arctan(1/\sqrt{2}$, $\mathcal{C}_{\rm N} > 0$) or negative ($\alpha > \arctan(1/\sqrt{2}$, $\mathcal{C}_{\rm N} > 0$) chirality.

This analysis is consistent with the criteria given in Supplementary Note 1. The determinant of the spiralization tensor (37) results in

$$\begin{aligned}\det\mathcal{D} &= -\frac{16D^2}{R^2A_\Omega^2}R_x^2\,R_y^2\ \cos(2\alpha) \\ &\quad -\frac{8D^2}{R^2A_\Omega^2}R_x\,R_y(R_y^2 - R_x^2)\ \sin(2\alpha)\,. \quad (40)\end{aligned}$$

The determinant is equal to zero, $\det\mathcal{D} = 0$, for angles

$$\alpha_o = \arctan\left(\frac{R_y}{R_x}\right) + n\cdot\frac{\pi}{2} \quad \text{with} \quad n\in\mathbb{Z}\,. \quad (41)$$

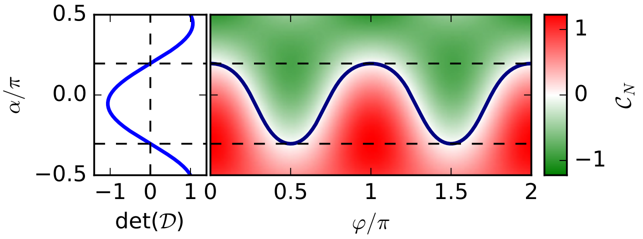

Supplementary Figure 3 | Néel-type chirality based on the micromagnetic model. Néel-type chirality (as colour code) for a bcc(110) surface as function of the relative angle α between the DM vector and the bond between atoms, and of φ parametrizing the propagation directions $\hat{\mathbf{e}}_\rho$. Regions of positive and negative chirality are separated by a solid line. For a broad range of $-0.3\pi \lesssim \alpha \lesssim 0.2\pi$ (indicated by horizontal dashed lines) multichiral skyrmion states are possible. In this range, the determinant of the spiralization tensor, $\det\mathcal{D}$, is negative. Values for $\mathcal{C}_{\rm N}$ have been scaled by a factor $\sqrt{3}/(aD)$ and $\det\mathcal{D}$ by $A_\Omega^2 4\sqrt{3}/(a^3D)$.

This reproduces the previous results $\alpha_o = -\arctan(\sqrt{2})$ and $\alpha_o = \arctan(1/\sqrt{2})$ for values of R_x and R_y given by the bcc(110) lattice. In between those two values, $\det\mathcal{D}$ becomes *negative* (see Supplementary Fig. **3**) preferring consistent with the conditions derived in the Supplementary Note 1 the antiskyrmion over the skyrmion. Taking into account our definition of the angle α, α_o denotes DM vectors pointing parallel to one of the mirror planes of the system, i.e. parallel to the x- or y-axis of the coordinate system shown in Supplementary Fig. **2**. This can easily be seen for the special case of $R_x = R_y$ (i.e. a square lattice) where one obtains $\alpha_o = \frac{\pi}{4} + n\cdot\frac{\pi}{2}$. However, one should keep in mind, if $R_x = R_y$ then usually this change is related to a structural transition from C_{2v} to C_{4v} symmetry where additional mirror symmetries (see arguments in main text) only allow $\alpha_{C_{4v}} = \frac{\pi}{2} + n\pi$. Thus, the case $\det\mathcal{D} = 0$ is forbidden and furthermore it follows $\det\mathcal{D} = -4D^2R^2\cos(2\alpha_o)/A_\Omega^2 = 4D^2R^2/A_\Omega^2 > 0$ leading only to skyrmions.

Supplementary Note 5 | Computational details of DFT calculations for 2Fe/W(110)

We performed DFT calculations employing the non-collinear version[10] of the full-potential linearized augmented plane-wave method[11,12] (FLAPW) in film geometry as implemented in the `FLEUR` code (see www.flapw.de). The structural properties such as the lattice constant and the interlayer relaxation were taken from Heide *et al.*[13]. An asymmetric slab consisting of seven W layers and two Fe layers was used as structural

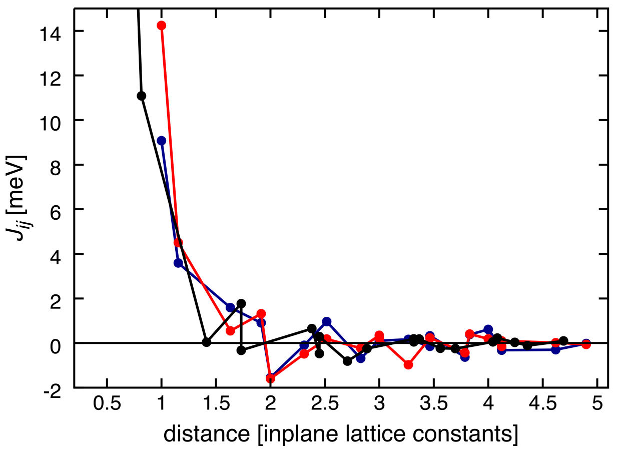

Supplementary Figure 4 | Exchange parameters J_{ij} in 2Fe/W(110). Calculated exchange constants J_{ij} for the intralayer coupling in the surface Fe layer (blue), the intralayer coupling in the interface Fe layer (red) and the interlayer interaction between both layers (black).

model. A self-consistent calculation without spin-orbit coupling was carried out for the ferromagnetic state with 1600 k-points in the full two-dimensional (2D) Brillouin zone (BZ) and a plane-wave cutoff of 4.2 bohr^{-1} serving as starting point for the non-collinear calculations to follow. An exchange correlation potential in local density approximation[14] was used.

Subsequently, calculations of the electronic structure and total energy were performed for spin-spiral states for a set of wave vectors $\mathbf{q}$. Thus, for a spin-spiral the magnetic moment vector, $\mathbf{M}$, at site $\mathbf{R}$ is determined by

$$\mathbf{M}(\mathbf{q}) = M\ \mathcal{R}(\hat{\mathbf{n}})\ \mathbf{S}(\mathbf{q}) = M\ \mathcal{R}(\hat{\mathbf{n}}) \begin{pmatrix} \sin\theta\ \cos(\mathbf{R}\cdot\mathbf{q}+\phi) \\ \sin\theta\ \sin(\mathbf{R}\cdot\mathbf{q}+\phi) \\ \cos\theta \end{pmatrix}, \quad (42)$$

where $\mathcal{R}(\hat{\mathbf{n}})$ is a rotation matrix that rotates the rotation axis z of the above equation into the actual rotation axis $\hat{\mathbf{n}}$ around which the spin-spiral rotates. ϕ enables phase differences in the spirals between the first and second layer. The cone angle θ measures the difference between the spin-spiral and the ferromagnetic state.

In order to speed up the calculations the force theorem[15,16] is used, meaning, the self-consistently obtained charge density of the ferromagnetic state can be used to calculate the properties of any spin-spiral state with a wave vector $\mathbf{q}$ and then the effect of spin-orbit coupling (SOC) is added in first-order perturbation[17] for any state $\mathbf{q}$. For this step, the number of $\mathbf{k}$-points was increased to 4096 to improve the accuracy as a small cone angle results in small energy differences. To calculate the inter- and intralayer DM vectors and exchange constants, the total energy with and without SOC was calculated on an 8×8 $\mathbf{q}$-point mesh in the 2D BZ. The real-space parameters were obtained via Fourier-transformation. To obtain accurate model parameters of this system around its ground state (i.e. the FM) a small cone angle θ of 5° was used.

By fixing the magnetic moments of one of the Fe layers (and the non-magnetic substrate) to ferromagnetic alignment and thus letting the spin-spiral propagate only in the remaining layer we are able to determine the intralayer DM interaction, whereas if the spin-spiral is allowed into both layers simultaneously the interlayer DM interaction can be calculated. The calculated exchange parameters can be found in Supplementary Fig. **4**. We observe the expected decay of J_{ij} with distance $|\mathbf{R}_i - \mathbf{R}_j|$, although one should keep in mind that the number of parameters increase linear with distance. On top we find oscillations of size and sign of J_{ij} as function of direction resulting from the very anisotropic 2D Fermi surface.

Supplementary Note 6 | Analysis of DM energy contributions to stability of antiskyrmion in 2Fe/W(110)

To analyse in more detail the role of different DM energy contributions to the stabilization of the antiskyrmion, we plotted in Supplementary Fig. **5** at each site i the local DM energy density by summing over all sites j of the intra- and interlayer DM interactions, i.e. within and between the two layers, respectively, evoking the spin-lattice description of the DM energy in Eq. (1) of the main text with parameters summarized in Fig. 3(d) of the main text and Supplementary Fig. **4**. By inspection of Supplementary Fig. **5**, it becomes obvious, that the main stabilization of the antiskyrmion stems from the contributions within the layers and not between the layers and the dominating contribution comes from the core of the antiskyrmion due to the intralayer DM interaction in the Fe interface layer (see red area in the upper right panel of Supplementary Fig. **5**). However, the rotational sense of the magnetization texture along the [001] (i.e. y-) direction of the lattice (the spins positioned in Supplementary Fig. **5** "north" and "south" of the center of the antiskyrmion) is energietically disfavoured by the underlying magnetic interaction. This energy loss is nevertheless compensated by the energy gain of the surface layer along those directions and thus in total, the antiskyrmion gains energy along all crystallographic directions.

As already noticed in Fig. 3 of the main text, the rotational sense enforcing component of the DMI, $(\mathbf{D}_{ij})_\varphi$, of the Fe layer showed changing signs for different neighbours and was mainly satisfied with the formation of the antiskyrmion. Therefore, we carried out spin dynamics simulations where we only included the intralayer DM

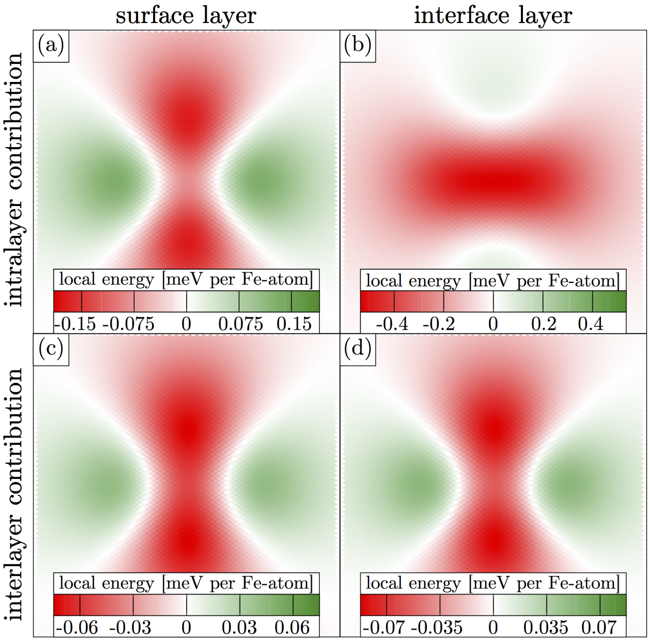

Supplementary Figure 5 | Local energy distributions of the DMI in 2Fe/W(110). Shown are the intra- and interlayer contributions of DMI of the Fe surface and interface layer in 2Fe/W(110) for the antiskyrmion shown in Fig. 4 of the main article. A negative (red) (positive (green)) energy density indicates a local rotational sense that is energetically favoured (disfavoured). An area of 20 nm × 20 nm around the center of the antiskyrmion is shown. Notice the different colour scales of the different panels.

interactions of the interface layer to check whether then an antiskyrmion could indeed be stable with those parameters. However, we found this is not the case. The antiskyrmion spreads along the y-axis (in agreement with the previously shown analysis) and thus results into a stripe domain wall. On the other hand we realized that the interlayer contributions of the DMI are larger than the interlayer contributions and indeed if we switch off in the spin-dynamics calculation the interlayer contribution, the antiskyrmion is stable. This shows, that for 2Fe/W(110) in particular the interplay of all intralayer DM interactions are crucial for the possible formation of stable antiskyrmions in this system.

Supplementary Note 7 | Dzyaloshinskii-Moriya vectors for 2Fe/W(110) from the Korringa-Kohn-Rostoker Green-function method

Since the FLAPW approach to calculate microscopic atom-pair dependent DM parameters $\mathbf{D}_{ij}$ used in the main text was newly developed and used here for the first time, for comparison we list in Supplementary Table **1** in addition the $\mathbf{D}_{ij}$ vectors calculated by means of the Korringa-Kohn-Rostoker Green-function (KKR-GF) method[18]. Using tke KKR-GF method we first converge the DFT potential including spin-orbit coupling self-consistently, with a chosen magnetization along the z-direction, and then perform three one-shot calculations with magnetizations along the x, y and z axes, employing the relativistically generalized version[19,20] of the method of infinitesimal rotations[16], in order to access all three components of the DM vector.

The KKR-GF and FLAPW calculations are performed with the same structural parameters and the same exchange-correlation functional[14]. The numerical parameters chosen include an angular-momentum cutoff $\ell_{\max} = 3$, a grid of 50 points for integrations along a complex-energy contour with a Fermi smearing of 473 K and 1600 (6400) k points in the full 2D BZ for the integration of the Matsubara pole closest to the real energy axis for self-consistency (in the one-shot calculations employing infinitesimal rotations).

Supplementary Table 1 | Comparison of Results between KKR-GF and FLAPW method Microscopic Dzyaloshinskii-Moriya vectors $\mathbf{D}_{ij}$ calculated with two different methods for a representative pair of atoms for the first three shells within the surface layer (S), the interface layer (I), or between both (IS). The real-space vectors connecting the atoms, $\mathbf{R}_{ij}$, are given in units of the W-bulk lattice constant $a_{3D} = 0.316$ nm, and $d = 0.54152\,a_{3D}$ is the interlayer distance.

	$\mathbf{R}_{ij}$	$\mathbf{D}_{ij}$ [meV] KKR	FLAPW
S	$(\frac{1}{\sqrt{2}}, \frac{1}{2}, 0)$	$(-0.389, 1.064, 0)$	$(-0.420, 1.062, 0)$
	$(0, 1, 0)$	$(0.159, 0, 0)$	$(0.082, 0, 0)$
	$(\sqrt{2}, 0, 0)$	$(0, -0.177, 0)$	$(0, 0.005, 0)$
I	$(\frac{1}{\sqrt{2}}, \frac{1}{2}, 0)$	$(0.837, -0.639, 0)$	$(0.769, -0.732, 0)$
	$(0, 1, 0)$	$(-0.632, 0, 0)$	$(-0.604, 0, 0)$
	$(\sqrt{2}, 0, 0)$	$(0, -0.270, 0)$	$(0, -0.220, 0)$
IS	$(0, \frac{1}{2}, d)$	$(-0.044, 0, 0)$	$(0.013, 0, 0)$
	$(\frac{1}{\sqrt{2}}, 0, d)$	$(0, 0.059, 0)$	$(0, 0.116, 0)$
	$(\frac{1}{\sqrt{2}}, 1, d)$	$(-0.283, 0.018, -0.134)$	$(-0.336, 0.044, -0.154)$

The Supplementary Table **1** reveals an overall good agreement at the sub-meV level between the DM vectors calcuated by KKR and FLAPW. Test simulations comparing the different sets of DM vectors and exchange parameters disclose the same picture of stable antiskyrmions although the exact diameter of the antiskyrmion and/or the required magnetic field may vary slightly. This is consistent with our finite sub-meV resolution and the number of shells we take into account in our calculations.

A detailed discussion of the two methods employed to

extract the $\mathbf{D}_{ij}$ vectors within the different formalisms, i.e. KKR-GF and FLAPW, and a comparison of the results will be published elsewhere.

Supplementary References

[1] Melcher, C. Chiral skyrmions in the plane. *Proc. R. Soc. A* **470,** 20140394 (2014).

[2] Belavin, A.A. & Polyakov, A.M. Metastable states of two-dimensional isotropic ferromagnets. *JETP lett* **22,** 245–248 (1975).

[3] Lin, F. & Yang, Y. Existence of two-dimensional skyrmions via the concentration compactness method. *Comm. Pure Appl. Math.* **57,** 1332–1351 (2004).

[4] Barrera, R. G., Estevez, G. A. & Giraldo, J. Vector spherical harmonics and their application to magnetostatics. *Eur. J. Phys.* **6,** 287–294 (1985).

[5] DeSimone, A., Kohn, R. V., Müller, S., & Otto, F. Recent analytical developments in micromagnetics in *The Science of Hysteresis*, Bertotti, G. & I. D. Mayergoyz, I. D., eds., vol. 2 of Physical Modelling, Micromagnetics, and Magnetization Dynamics, Academic Press, Oxford 2(4), 269–381 (2006).

[6] Bogdanov, N. & Hubert, A. Thermodynamically stable magnetic vortex states in magnetic crystals. *J. Magn. Magn. Mater.* **138,** 255–269 (1994).

[7] Nagaosa, N. & Tokura, Y. Topological properties and dynamics of magnetic skyrmions. *Nature Nanotech.* **8,** 899–911 (2013).

[8] Döring, L., Melcher C., Compactness results for static and dynamic chiral skyrmions near the conformal limit. *Calc. Var. Partial Differential Equations* **56,** 60 (2017).

[9] Hinzen, M. *Magnetisierungsfelder in ferromagnetischen Kristallen mit Dzyaloshinskii-Moriya-Wechselwirkung*, Master thesis, RWTH Aachen (2015).

[10] Kurz, Ph., Förster, F., Nordström, L., Bihlmayer, G., & Blügel, S. *Ab initio* treatment of non-collinear magnets with the full-potential linearized augmented planewave method. *Phys. Rev. B* **69,** 024415 (2004).

[11] Wimmer, E., Krakauer, H., Weinert M. & Freeman, A. J. Full-potential self-consistent linearized-augmented-plane-wave method for calculating the electronic structure of molecules and surfaces: O_2 molecule. *Phys. Rev. B* **24,** 864 (1981).

[12] Weinert M., Wimmer, E. & Freeman, A. J. Total-energy all-electron density functional method for bulk solids and surfaces. *Phys. Rev. B* **26,** 4571 (1982).

[13] Heide, M., Bihlmayer, G. & Blügel, S. Dzyaloshinskii-Moriya interaction accounting for the orientation of magnetic domains in ultrathin films: Fe/W(110). *Phys. Rev. B* **78,** 140403(R) (2008).

[14] Moruzzi, V. L., Janak, J. F. & Williams, A. R. *Calculated Electronic Properties of Metals*, Pergamon Press, New York (1978).

[15] Mackintosh, A. R., Andersen, O. K., *Electrons at the Fermi Surface*, ed. M. Springford (1980).

[16] Liechtenstein, A. I., Katsnelson, M. I., Antropov, V. P. & Gubanov, V. A. Local spin density functional approach to the theory of exchange interactions in ferromagnetic metals and alloys. *J. Magn. Magn. Mater.* **67,** 65–74 (1987).

[17] Heide, M., Bihlmayer, G., Blügel, S., Describing Dzyaloshinskii-Moriya spirals from first principles. *Physica B* **404,** 2678–2683 (2009).

[18] Bauer, D. S. G. *Development of a relativistic full-potential first-principles multiple scattering Green function method applied to complex magnetic textures of nano structures at surfaces*, Ph.D. thesis, RWTH Aachen (2013).

[19] Udvardi, L., Szunyogh, L., Palotás, K. & Weinberger, P. First-principles relativistic study of spin waves in thin magnetic films. *Phys. Rev. B* **68,** 104436 (2003).

[20] Ebert, H. & Mankovsky, S. Anisotropic exchange coupling in diluted magnetic semiconductors: *Ab initio* spin-density functional theory. *Phys. Rev. B* **79,** 045209 (2009).
